# A Network Perspective on Neuropsychiatric and Cognitive Symptoms of the Post-COVID Syndrome

**DOI:** 10.5964/ejop.10097

**Published:** 2022-11-30

**Authors:** Daniel Scharfenberg, Ann-Katrin Schild, Clemens Warnke, Franziska Maier

**Affiliations:** 1 Medical Psychology | Neuropsychology & Gender Studies, Center for Neuropsychological Diagnostic and Intervention (CeNDI), Faculty of Medicine and University Hospital Cologne, University of Cologne, Cologne, Germany; 2 Department of Psychiatry, Faculty of Medicine and University Hospital Cologne, University of Cologne, Cologne, Germany; 3 Department of Neurology, Faculty of Medicine and University Hospital Cologne, University of Cologne, Cologne, Germany; Dublin City University, Dublin, Ireland

**Keywords:** Long COVID, network model, brain fog, cognitive deficits, SARS-CoV-2, fatigue, post-vac syndrome

## Abstract

Many patients that were infected with SARS-CoV-2 experience cognitive and affective symptoms weeks and months after their acute COVID-19 disease, even when acute symptoms were mild to moderate. For these patients, purely neurological explanations are struggling to explain the development and maintenance of the great variety of neuropsychiatric and cognitive symptoms occurring after COVID-19. We provide a psychological perspective based on the network theory of mental disorders as an added explanation that does not displace neurological mechanism but rather complements them. We suggest viewing the SARS-CoV-2 infection as a trigger that first activates nodes in a causally connected network of neuropsychiatric and cognitive symptoms. In the following, activation will spread throughout the network that will get in a self-sustaining stable and dysfunctional state manifesting in ongoing symptoms known as post-COVID-19 syndrome. The network perspective allows to generalize explanations for persistent neuropsychiatric and cognitive symptoms to patients that experienced mild or moderate acute courses of COVID-19, but also to similar phenomena following other viral infections. In addition, it could explain why some symptoms did not occur during acute COVID-19, but develop weeks or months after it. This network perspective shifts the focus from viewing persistent symptoms as a continuation of COVID-19 to acknowledging it as a complex syndrome that indeed originates from the disease but fully unfolds after it (post-COVID). To test the presented network perspective, we will need extensive cross-sectional as well as longitudinal data on cognitive and neuropsychiatric symptoms in post-COVID patients.

Many individuals who were infected with SARS-CoV-2 experience symptoms of illness even weeks and months after their acute COVID-19 disease. This phenomenon has been referred to as ongoing symptomatic COVID-19, post-acute COVID-19, long-COVID syndrome or post-COVID syndrome, with varying definitions of the latter terms in the current literature. The NICE guideline suggests using the term “post-COVID-19 syndrome” for “signs and symptoms that develop during or after an infection consistent with COVID-19” and are “present for more than 12 weeks and are not attributable to alternative diagnoses” (National Institute for Health and Care Excellence, 2020). The post-COVID syndrome does not only affect patients with severe course of the disease, namely those who were hospitalized or ventilated (Bellan et al., 2021), but also patients who had mild to moderate symptoms and stayed at home or even experienced asymptomatic infections (Augustin et al., 2021). A recent meta-analysis estimated that up to 43% of people experience persistent symptoms after COVID-19 (Chen et al., 2022).

Patients with post-COVID syndrome report varying symptoms including pulmonary, hematologic, renal, endocrine, gastrointestinal or dermatologic manifestations (Nalbandian et al., 2021). In addition, patients often report neuropsychiatric and cognitive symptoms. A recent meta-analysis and systematic review estimated that a substantial proportion of persons experience symptoms like sleep disorders (24%), depression (14%), anxiety (21%) and fatigue (32%) 3 to 6 months after having been infected with SARS-CoV-2. Cognitive symptoms (36%) include difficulties to concentrate, brain fog or confusion (Alkodaymi et al., 2022). Two recent reviews found cognitive impairments in patients with the post-COVID syndrome in all neurocognitive domains, namely memory and learning, attention, executive functioning, language, and visual perceptive functions, with a focus on deficits in memory and learning, attention and executive functioning (Bertuccelli et al., 2022; Daroische et al., 2021). A study that extensively assessed cognitive function solely in patients that experienced asymptomatic or mild to moderate acute COVID-19 symptoms obtained similar results, finding impairments in all assessed cognitive domains that are learning and memory, complex attention, executive function, language and perceptual-motor function (Schild et al., 2022). Similar neuropsychiatric symptoms were also reported in SARS-CoV-1 and MERS-CoV, two other viruses from the corona virus family (Rogers et al., 2020). However, it is still unclear how these symptoms develop and persist over time.

So far, explanations for these symptoms as part of the post-COVID syndrome focused on neurological or neuroimmunological causes. Potential neurological mechanisms that lead to cognitive and neuropsychiatric symptoms include viral persistence and infiltration of the central nervous system (Mehandru & Merad, 2022; Troyer et al., 2020). However, analyses of the cerebrospinal fluid that show no viral RNA in post-COVID patients and no hint for chronic SARS-CoV2-directed intrathecal IgG response with neurological symptoms do not support the hypothesis of persistent infection of the central nervous system as a cause of neurological and neuropsychiatric symptoms (Schweitzer et al., 2022). Other potential neurological mechanisms include cytokine network dysregulation, peripheral immune cell transmigration, post-infectious autoimmunity or immunomodulatory treatments (Troyer et al., 2020). To date, these neurological hypotheses could not provide direct evidence to fully explain the development, maintenance and diversity of observed cognitive and neuropsychiatric symptoms in post-COVID syndrome.

## Hypothesis

We will provide an interdisciplinary network perspective based on the network theory of mental disorders (Borsboom, 2017) that could possibly explain persistent psychiatric and cognitive symptoms in patients after mild to moderate course of COVID-19. Intriguingly, such hypothesis would not be exclusive, and may add to other possible elements that contribute to pathogenesis, such as viral persistency or secondary autoimmunity discussed above. The network theory of mental disorders would provide an (added) explanation for post COVID-19, but also for similar observations after other viral infections of the past, such as following the MERS-CoV and SARS-CoV pandemics (Rogers et al., 2020).

During the last years, the network theory of mental disorders gained increasing reputation as an alternative conceptualization of mental disorders opposed to categorical classification systems. It considers mental disorders as syndromes, i.e. constellations of symptoms that occur together. In its core, the theory does not rely on aetiologies but rather states that symptoms are causally connected to other symptoms.

These interactions between symptoms can be portrayed in a network, in which nodes depict symptoms while the edges between nodes depict causal relationships. An edge between two symptoms means that a change in the activation of one node leads to a change in the activation of another node. These connections could arise inter alia due to biological or psychological mechanisms. Additionally, nodes, or symptoms, can be influenced by factors that stand outside the network system in the so-called “external field”. Examples for this kind of network could be critical life events or somatic conditions like inflammation processes or viral infections (Borsboom, 2017).

**Figure 1:**
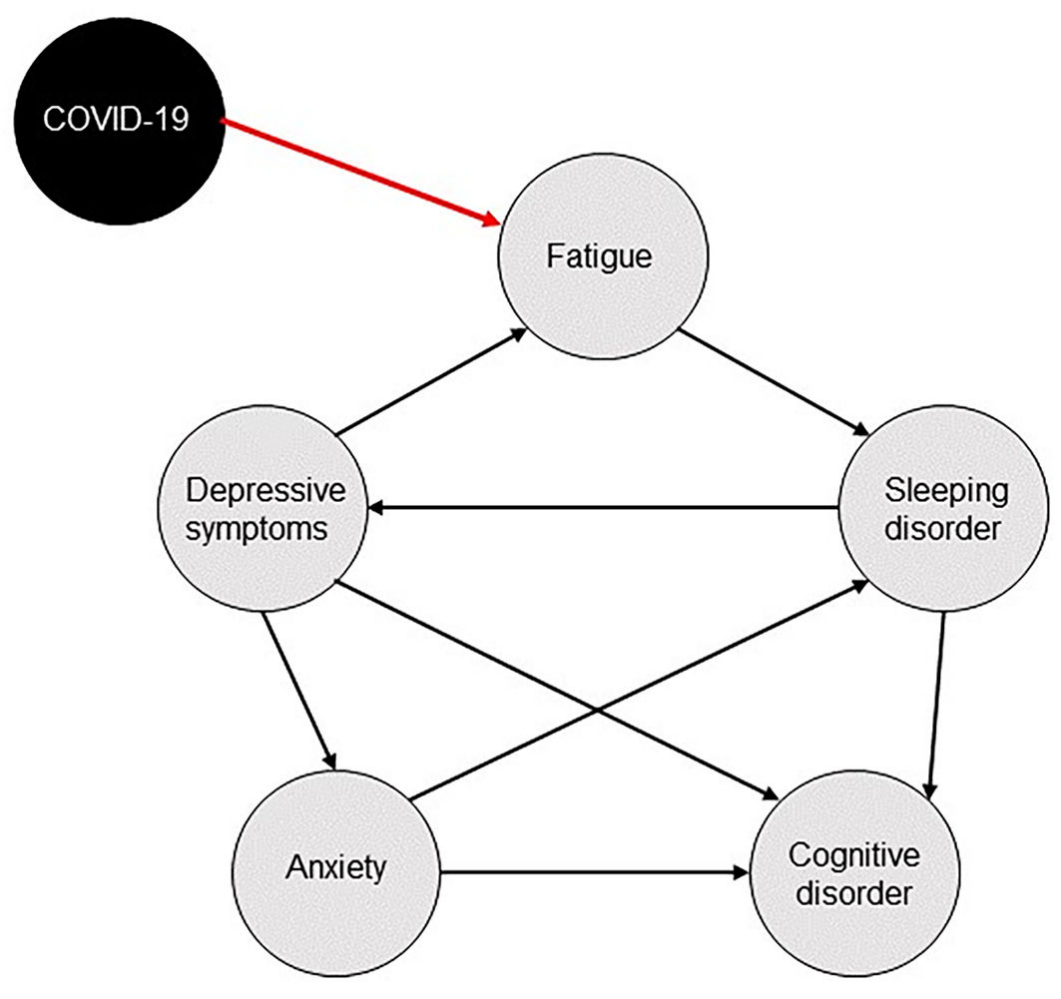
Possible Pathway of Activation in the Post-COVID Network of Neuropsychiatric and Cognitive Symptoms *Note.* Acute COVID-19 infection in the external field (black node) initially activates symptoms, e.g., fatigue, that spread the activation to other symptoms in the network (grey nodes).

Although COVID-19 has a clear medical aetiology, the SARS-CoV-2 virus, it is still possible to apply the network theory of mental disorders to neuropsychiatric and cognitive symptoms of the post-COVID syndrome. That is, if we view the viral infection as a factor in the external field that causes initial activation of nodes in the symptom network. For example, it is possible that the infection could cause initial symptoms like fatigue and tiredness. From these initial symptoms, activation will spread throughout the network. Figure 1 displays a possible pathway of activation. In this example, fatigue may lead to disrupted sleep, which in turn could lead to tiredness and finally induces impaired cognitive performance or depressive symptoms that are connected with the anxiety symptom cluster. Note that this is one of many possible causal pathways in the network and edges in individual symptom networks may even differ between persons.

However, the question remains as to why symptoms persist even after the source of initial activation is already gone. To explain this phenomenon, we need to apply the principle of hysteresis that is described in the network theory of mental disorders. It “implies that symptoms continue to activate each other, even after the triggering cause of the disorder has disappeared” (Borsboom, 2017, p. 9). To apply this principle, the network needs to be strongly interconnected. Then, it results in a self-sustaining network, as feedback interactions between nodes do not lead to a loss in activation. By this, the network remains in a stable but dysfunctional state with activated neuropsychiatric and cognitive symptoms.

Relevant nodes of neuropsychiatric symptoms in this network could be fatigue, anxiety and depressive symptoms, disturbed sleep, distress and cognitive impairment but may also be connected to neurological symptoms in the external field, e.g. myalgia or neuroinflammatory processes.

## Discussion

The network theory of mental disorders can be a useful approach to explain psychiatric and cognitive symptoms in post-COVID syndrome. It has some advantages over other offered explanations.

As already mentioned, the principle of hysteresis can explain why neuropsychiatric post-COVID symptoms are so stable over the time, even when the virus is no longer apparent in the body (Schweitzer et al., 2022). Additionally, the network perspective can explain the phenomenon that some post-COVID symptoms do not persist following the acute infection but rather newly develop afterwards (National Institute for Health and Care Excellence, 2020). This explanation could change our view (at least for neuropsychiatric and cognitive symptoms) on the syndrome not to be the prolongation of the viral disease, as it is reflected by the term “Long-COVID” which is often used in ongoing science communication, but rather a separate condition that only originates in the acute infection with SARS-CoV-2, as it is reflected in the term “post-COVID”.

This especially applies to patients who experienced a mild course of acute COVID-19 but developed post-COVID symptoms nonetheless. The network perspective is able to explain this phenomenon by arguing that strength and stability of causal relationships between symptoms (i.e., edges) are more important for spreading activation in a network than the severity of the initial symptom (i.e., activation of the node), e.g., the feeling of fatigue and illness experienced during the acute phase of COVID-19.

Another advantage is the generalizability of this approach. This network perspective cannot only explain symptoms of the post-COVID syndrome, but also similar phenomena occurring after different viral infections as observed in infections with other corona viruses like MERS-CoV and SARS-CoV (Rogers et al., 2020). It could possibly even explain cognitive and neuropsychiatric symptoms similar to those observed in the post-COVID syndrome following other viral or bacterial infections, e.g. in infections with influenza, the West-Nile virus (Damiano et al., 2022), the Hepatitis-C virus (Monaco et al., 2015) or multi-bacterial infections that cause periodontitis (Hashioka et al., 2019).

To test applications of the network theory on the post-COVID syndrome, future research should collect not only cross-sectional but also longitudinal data on neuropsychiatric and cognitive symptoms in patients that were infected with SARS-CoV-2. The method of network analysis will help to identify potential causal associations between symptoms that could contribute to explain persistent neuropsychiatric symptoms of COVID-19 and show how post-COVID networks evolve and maintain over the course of time (Borsboom et al., 2021; Borsboom & Cramer, 2013). There already are some studies that applied network analyses to COVID-19 symptom networks (Liu et al., 2021; Taquet et al., 2021). Therefore, future studies should apply network analysis based on the perspective provided in this article to test the hypothesis that neuropsychiatric and cognitive symptoms of the post-COVID syndrome can be conceptualized within the network theory of mental disorders. Confirmatory analyses could build on the extensive body of literature that provides evidence of at least correlational relationships between neuropsychiatric and cognitive symptoms. It is well known that several neuropsychiatric symptoms are not independent, e.g. that depressive and anxiety disorders have common symptoms connecting their network symptom clusters (Beard et al., 2016; Kaiser et al., 2021) or that both of them are associated with sleeping disorders (Becker et al., 2017; Cox & Olatunji, 2020). Furthermore, neuropsychiatric symptoms often occur together with cognitive disorders (Martin & Velayudhan, 2020). Specifically, there is evidence for associations between cognition and depression (Anapa et al., 2021; Rock et al., 2014), anxiety (Gulpers et al., 2016) or sleeping disorders (Fortier-Brochu et al., 2012; Miyata et al., 2013). To overcome the challenge of only describing bivariate correlations among possibly relevant symptoms in post-COVID syndrome, causal relationships from symptom networks have to be identified by collecting longitudinal data on various neuropsychiatric and cognitive symptoms in post-COVID patients.

## Conclusion

The provided network perspective on neuropsychiatric and cognitive symptoms could help to better understand development, maintenance and diversity of persistent symptoms observed in patients with the post-COVID syndrome. This approach explains the post-COVID syndrome equally well for patients who experienced severe courses of acute COVID-19 as well as for patients with mild to moderate acute COVID-19 symptoms. Additionally, it offers the possibility to explain similar symptoms occurring after other types of viral infections, or even reports of post-COVID-like symptoms following vaccination (post-vac syndrome) that recently arose in present science communication (Couzin-Frankel & Vogel, 2022). Being a psychological approach to explain post-COVID symptoms, it provides an added interdisciplinary understanding of post-COVID syndrome. It complements prevailing neurological theories that were not able to fully explain the syndrome yet, because the causal relationships between symptoms or the initial activation of symptoms in the network may be best explained by neurological mechanisms, e.g. inflammation processes in the central nervous system during acute infection with SARS-CoV-2 or neurological structural or functional changes that could explain associations between neuropsychiatric symptoms. Thus, to fully understand the causes and maintenance of neuropsychiatric and cognitive symptoms of the post-COVID syndrome, psychological, neuropsychiatric and neurological approaches should be applied likewise.
